# Rootstock-Scion Interaction Affects the Composition and Pathogen Inhibitory Activity of Tobacco (*Nicotiana tabacum* L.) Root Exudates

**DOI:** 10.3390/plants9121652

**Published:** 2020-11-26

**Authors:** Cheng-Sheng Zhang, Yanfen Zheng, Lijuan Peng, Jianmin Cao

**Affiliations:** 1 Tobacco Research Institute of Chinese Academy of Agricultural Sciences, Qingdao 266101, China; zhengyanfen@caas.cn; 2 Special Crops Research Center of Chinese Academy of Agricultural Sciences, Qingdao 266101, China; 3 Yunnan Tobacco Quality Supervision and Test Station, Kunming 650106, China; pengljkm2003@hotmail.com

**Keywords:** *Phytophthora nicotianae*, allelopathy, disease resistance mechanism, grafting

## Abstract

The composition and allelopathy to *Phytophthora nicotianae* (the causal agent of tobacco black shank disease) of root exudates from a resistant tobacco (*Nicotiana tabacum* L.) cultivar Gexin 3, a susceptible cultivar Xiaohuangjin 1025 and their reciprocal grafts were investigated. Grafting with disease-resistant rootstock could improve resistance to black shank; this is closely related to the allelopathy of root exudates. The root exudates from the resistant cultivar inhibited the growth of *P. nicotianae*, while those from the susceptible cultivar promoted the growth; the grafting varieties had intermediate properties. The root exudate composition differed among cultivars. Gexin 3 was rich in esters and fatty acids, while Xiaohuangjin 1025 contained more hydrocarbons and phenolic acids. The composition of root exudates of grafted cultivars as well as their allelopathy to *P. nicotianae* were altered, and tended to be close to the composition of cultivar used as rootstock. Eugenol, 4-tert-butylphenol, mono (2-ethylhexyl) phthalate, 4-hydroxybenzoic acid, 2,6-di-tert-butylphenol, dipropyl phthalate, and methyl myristate were identified as the main compounds contributing to inhibitory properties of root exudates. Sorbitol was suggested to play a role in disease induction. Overall, rootstock–scion interaction affected the composition of tobacco root exudates, which may be attributed to the different disease resistance among grafted plants, rootstock and scion.

## 1. Introduction

Tobacco black shank, caused by the oomycete pathogen *Phytophthora nicotianae*, is a destructive disease with worldwide distribution. Because of the wide range of crop hosts, the diversity of disease transmission, and the formation of oospores and other dormant bodies which can survive in the soil for long periods, as well as the low resistance of major cultivated tobacco (*Nicotiana tabacum*) varieties, there are no effective prevention and control measures at present [1,2]. In commercial production, chemical agents such as metalaxyl mancozeb, propamocarb and dimethomoph are often used for disease control. However, the widespread use of these agents has many negative effects such as environmental pollution, fungicide residues in crops, and increased pathogen resistance [3].

Grafting with disease-resistant rootstock is considered to be an effective way to control plant soil-borne diseases, and has been widely applied in the production of fruits, vegetables, cotton, and other economic crops [4,5,6,7]. Apart from increasing disease resistance, grafting can also enhance plant stress resistance, promote growth and improve some traits [8]. So far, there have only been a few studies on the control of tobacco black shank using grafting. Independent studies performed by Wang et al. [9] and Liu et al. [10] demonstrated that susceptible tobacco grafted onto disease-resistant rootstock could significantly reduce the disease severity of black shank. Therefore, further screening of grafting varieties and understanding the mechanism of disease resistance is important for tobacco black shank control.

However, there are few reports on the mechanism of grafting to improve plant disease resistance. It is generally believed to be associated with the change of morphological structure, physiological and biochemical responses, and the expression of disease-resistant genes [11,12,13]. Root exudates are a mixture of a variety of compounds, which were exuded to rhizosphere through root [14,15,16]. Previous studies have shown that root exudates could affect plant resistance through their direct allelopathic effects (positive or negative) on pathogens [17]. The components and functions of root exudates were different among crop cultivars including grafted cultivars. The species and relative contents of root exudate compounds from grafted eggplant were different from that from the original stock or scion [18]. The inhibition effect of watermelon root against *Fusarium oxysporum* f.sp. *niveum* was significantly enhanced after being grafted onto the rootstocks of disease-resistant cultivar [19]. Similar results have been reported for pepper, tobacco, and other crops [9,13]. These results motivated us to hypothesize that root exudates are involved in the enhanced disease resistance mechanism induced by grafting. To verify this, the composition of root exudates, and their allelopathic effects on *P. nicotianae* of a resistant tobacco cultivar, a susceptible tobacco cultivar, and their reciprocal grafting combinations (rootstocks and scions for each other) were investigated in this study. The results will provide a better insight into the role of root exudates in plant disease resistance.

## 2. Materials and Methods

### 2.1. Preparation of Test Plant Materials

The tobacco cultivars Gexin 3 (R) and Xiaohuangjin 1025 (S) were provided by the Chinese Tobacco Germplasm Resource Platform. The seeds of Gexin 3 and Xiaohuangjin 1025 were seeded in sterile soil. Grafting experiments were carried out at the rooting stage of seedling (with 6 true leaves). Gexin 3 and Xiaohuangjin 1025 served as rootstock and scion for each other, and were grafted by the splice grafting method [20]. After culturing for 10 days at 26 °C and 60% relative humidity, the survival grafting seedlings were selected for following studies. Two grafting varieties including rootstock/scion union of Gexin 3/Xiaohuangjin 1025 (RS) and Xiaohuangjin 1025/Gexin 3 (SR) were finally obtained. Grafted seedlings with the same growth status, as well as their stock parent and their scion parent were transplanted in vermiculite medium for seven days, and were used to collect root exudates. Plants were used to evaluate tobacco black shank resistance under pot experiment and field conditions according to Zhang et al. method [21]. Each treatment was repeated three times, and every replicate had 15 seedlings for each cultivar.

### 2.2. Preparation of Inoculum of Phytophthora nicotianae

*P. nicotianae* race 0 strain JM01 with high pathogenicity was obtained from the Integrated Pest Management Key Laboratory of China Tobacco, Qingdao, China [22]. *P. nicotianae* cultured on millet were used to field inoculation. Millets kernels were boiled until 2/3 of the husks were cracked, filtered with 2 layers of gauze, and aired to about 40% of water content (which can be held into a mass by hand but scattered when falling down). Then, it put it into a 500 mL of triangular flask and sterilize it at 121 °C for 20 min. After cooling, 3 agar plugs with diameter of 5 mm from five-day-old actively growing colonies were transferred to the millet medium, cultured at 26 °C for 15 d, and used within 24 h.

The zoospores of *P. nicotianae* were produced according to the method described in Zhang et al. [22]. Briefly, *P. nicotianae* was cultured for 21 days on an oat medium (OA) plate. Then, 0.1% KNO_3_ solution was added (10 mL per dish), followed by culturing at 26 °C for 72 h, and immediately chilled to 4 °C for 0.5 h. Spore suspension was carefully drawn, adjusted to 10^6^ colony-forming units (cfu) mL^−1^ by using a light microscope and a hemocytometer, and stored at −4 °C.

### 2.3. Disease Resistance Evaluation

The pot experiments were conducted in an artificial climatic chamber (26 °C, 60% relative humidity, light intensity 300 mmol m^−2^ s^−1^, 12 h light/12 h dark). Each seedling received 10 mL of *P. nicotianae* zoospore suspension (10^6^ spore m^−1^), and the disease severity was investigated after seven days of inoculation. Each treatment was repeated three times, and with each replicate containing 15 plants. The field tests were carried out at Tobacco Resources and Environment Scientific Experiment Station of Chinese Academy of Agricultural Sciences (Jimo, Qingdao) in 2016. Tobacco seedlings were transplanted on May 22th, and inoculated with *P. nicotianae* on July 10. The millets covered with mycelium were buried into 5 cm deep soil around stem base (5 g millet per plant). The disease severity was scored on July 25th. Each plot included 3 rows with a total of 45 plants. There were three replicates for each treatment. The disease index was calculated by the following formula:

$$\mathrm{Disease}\;\mathrm{index}\;=\frac{\left(a\times 0\right)+\left(b\times 1\right)+\left(c\times 3\right)+\left(d\times 5\right)+\left(e\times 7\right)+\left(f\times 9\right)}{\left(a+b+c+d+e+f\right)\times 9}\times 100,$$

where a, b, c, d, e, and f are the number of plants in each disease category.

### 2.4. Root Exudates Collection

Root exudates were collected based on the method of Badri et al. [23]. Briefly, seedlings were taken from the vermiculite medium, the solid particles adhering to the root were removed, and the plants were rinsed with deionized water. Then, each plant was immersed in 50 mL of distilled water for 12 h in dark. The process was repeated for four plants of each cultivar and each reciprocal graft. The above liquid cultures were merged (total 200 mL), and extracted three times with the same volume of dichloromethane. The organic phases (neutral and basic fractions) were concentrated under a vacuum (−83 °C, 1.0 Pa). The root exudates obtained were resuspended to 1 mL with methanol, dehydrated with sodium sulfate, and stored at −20 °C for use.

### 2.5. Effects of Root Exudates on Phytophthora Nicotianae

The effects of root exudates on *P. nicotianae* were evaluated by testing the effects of the exudates on the mycelium growth rate and spore germination [22]. *P. nicotianae* strain JM01 tested was preserved in our laboratory [22]. Root exudates were diluted to the final content 20 mg ml^−1^, filtered with a 0.22 µm millipore filtration membrane, and blended to sterilized OA (cooled to 45 °C) with the volume ratio of 1:1, followed by pouring into Petri dishes. To avoid bacterial contamination, 0.03 g of chloramphenicol was added to every 100 mL of medium (with final content of chloramphenicol 0.3 mg/mL). An agar plug from an actively growing colony of *P. nicotianae* (0.6 cm diameter) was placed at the center of the Petri dish, incubated at 26 °C, and the colony diameter was measured from 3 to 7 days by using the cross method. OA medium containing the same content of chloramphenicol served as the control. Each treatment was repeated three times.

An amount of 100 μL of spore suspension (diluted to 10^6^ cfu ml^−1^) was transferred to a concave slide. It was blended with an equal volume of root exudate with a final concentration equal to 0.05 g mL^−1^, and incubated at 26 °C in the dark with a relative humidity of 80%. After 10 h of incubation, the zoospore germination was examined under a microscope (Olympus CX41RF, Tokyo, Japan). About 200 spores were examined for each treatment, and germination rate was expressed as percentage of the total number of spores examined. There were three replicates for each treatment.

### 2.6. Gas Chromatography-Mass Spectrometer (GC-MS) Analysis of Root Exudates

The components of root exudates were identified by gas chromatography-mass spectrometry (GC-MS) (Agilent 7890a-5975c, Santa Clara, CA, USA). A mid-polarity HP-5 MS capillary column (30 mm × 0.25 mm × 0.25 μm) was used under the following conditions: inlet temperature of 250 °C, initial column temperature of 50 °C maintained for 2 min, then increased to 250 °C at 6 °C/min, and maintained at 250 °C for 15 min. The carrier gas was helium, with a flow rate of 1.0 mL/min, and an injection volume of 1.0 mL. Mass condition: electron bombardment ion source, electron energy of 70 eV, interface, the scanning range of M/Z 30–600 AMU, a scanning speed of 0.2 S, and an ion source temperature of 230 °C. The extraction solvents methanol and dichloromethane were used as a blank test under the same conditions.

### 2.7. Identification of Root Exudates Composition

The identification of root exudates components was performed by the calculated retention indices (RI) and comparing the obtained mass spectra with those in reference compounds available in the NIST14 library (National Institute of Standards and Technology, Gaithersburg, MD, USA). The RI were determined in relation to a homologous series of n-alkanes (C7–C30) under the same operating conditions. The relative percentage amounts of the separated compounds were calculated from the integration of the peaks in flame ionization detector (FID) chromatograms. The composition of different root exudates samples was displayed with a heatmap, and an unsupervised hierarchical cluster analysis (HCA) was performed to investigate the overall differences of sample distribution and chemical composition.

### 2.8. Effects of Compounds on Phytophthora nicotianae Mycelia Growth

A total of 34 compounds were isolated and identified. Fourteen compounds were selected for the evaluation of their effects on *P. nicotianae* mycelia growth. The compounds tested were dissolved by dimethyl sulfoxide (DMSO), diluted to the required concentration as follows with ultrapure water, and filtered with 0.22 µm millipore filtration membrane. The prepared compound solutions were added to an oats culture medium plate to ensure the final concentrations as 5 μg L^−1^, 50 µg L^−1^ and 500 µg L^−1^, respectively. Then, an agar plug from an actively growing colony of *P. nicotianae* (0.6 cm diameter) was placed in the center of the plate, and incubated in dark at 27 °C for 5 days. The colony diameter was measured by the cross method. The DMSO and distilled water served as the control. Each treatment included three plates with three replicates. Additionally, sorbitol can affect the osmotic potential of culture medium, which in turn influences *P. nicotianae* mycelium growth. Therefore, the effect of sorbitol on *P. nicotianae* mycelium growth was further assayed at 6.25 mmol/L, 12.5 mmol L^−1^, 25 mmol L^−1^, 50 mmol L^−1^, and 100 mmol L^−1^, for comparison to glucose, a compound with a similar osmotic potential. 

### 2.9. Data Statistics

Excel 2013 and DPS 7.05 software were used for data analysis. Differences between groups were tested by using one-way analysis of variance (ANOVA), followed by Tukey’s multiple comparison test. *p* < 0.05 indicates that the differences are statistically significant. The correlation analysis between disease index (pot experiment) and mycelium inhibition rate of root exudates was performed with Excel 2013.

## 3. Results

### 3.1. Variety Resistance Evaluation for Tobacco Black Shank

**Figure 1:**
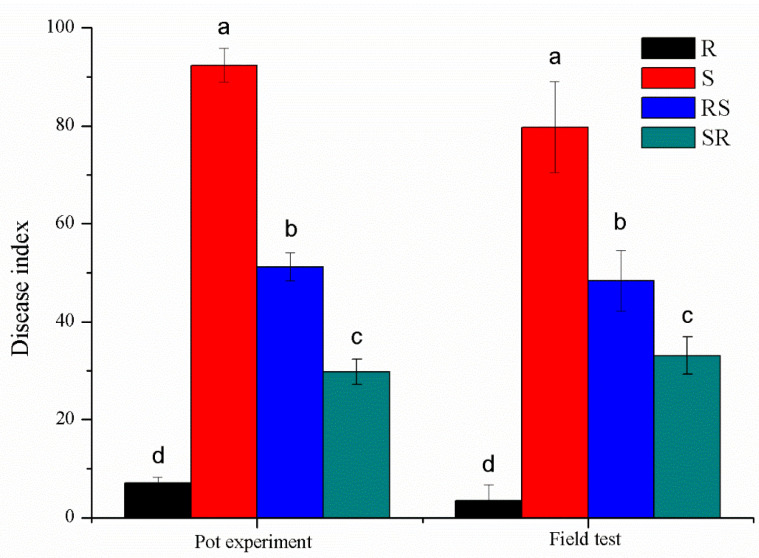
Susceptibility to tobacco black shank. R and S represent resistant and susceptible cultivars; SR and RS represent different scion/stock combinations; lower case letters indicate statistical differences within a column (*p* < 0.05); the error bars represent standard error of the mean.

The results of the pot experiment and field evaluation are shown in Figure 1. Although the disease severity was significantly different among the two cultivars and their grafting materials tested, each cultivar exhibited similar characteristics in both the seedling stage and the mature stage. Gexin 3 (R) showed high resistance with a disease index < 10, followed by the combination SR (with rootstock of R) which showed moderate resistance (less than 30 for the pot experiment, and about 30 in the field test). Xiaohuangjin 1025 (S) and its combination as rootstock (RS) displayed high susceptibility and moderate susceptibility, respectively. 

### 3.2. GC-MS Analysis of Root Exudates

**Figure 2:**
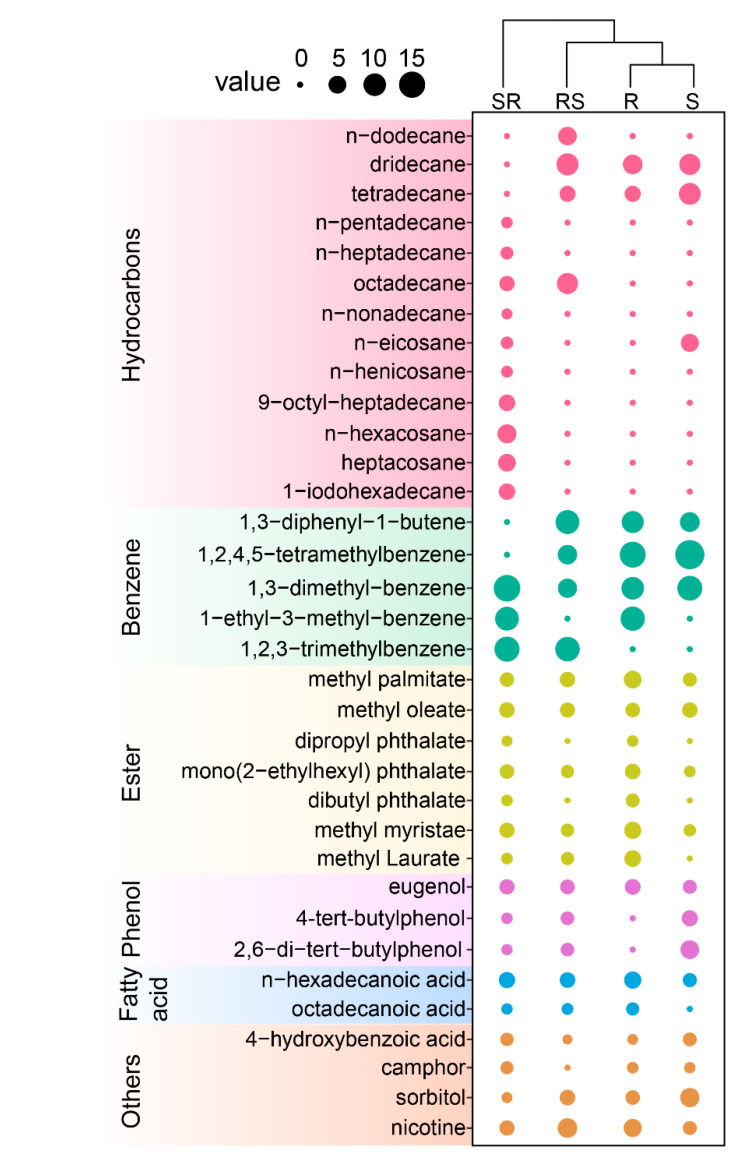
Chemical components of root exudates in different tobacco varieties and grafting combinations. R and S represent resistant and susceptible cultivars; SR and RS represent different scion/stock combinations; values indicate the peak area percentage obtained by GC-MS analysis.

**Figure 3:**
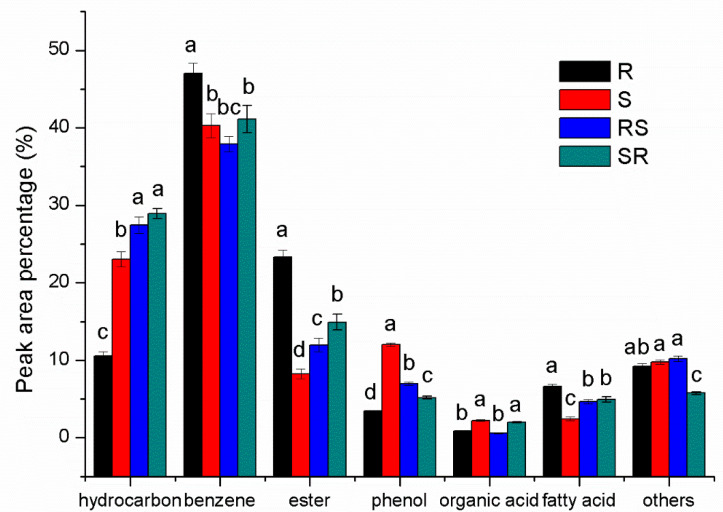
Peak area percentage of different kinds of compounds based on GC-MS analysis. R and S represent resistant and susceptible cultivars; SR and RS represent different scion/stock combinations; lower case letters indicate statistical differences within one column (*p* < 0.05); the error bars represent standard error of the mean.

A total of 34 compounds were identified from the root exudates of the four tobacco cultivars (Figure 2, Table S1). There were 14 hydrocarbons, 4 benzenes, 7 esters, 3 phenols, 2 fatty acids, 1 organic acid, 1 terpene, 1 alcohol and 1 alkaloid. Benzenes were the main components (accounting for 37.9–47.04%), followed by hydrocarbons (10.56.12–28.91%) (Figure 3). There were great differences in composition and relative contents of root exudates among cultivars assayed. Twenty compounds were detected in R root exudates, and 18 compounds were detected in S root exudates. There were significant differences in the composition of root exudates between resistant and susceptible varieties. Compared to S, the root exudates of R contained more esters (23.29% and 8.21% in R and S, respectively) and fatty acids (6.58% and 2.43% in R and S, respectively) but less hydrocarbons (20.12% and 30.12% in R and S, respectively) and phenolic acids (4.29% and 14.25% in R and S, respectively) (Figure 2). Obviously, grafting altered the composition of exudates as supported by hierarchical cluster analysis (HCA) analysis (Figure 2), where R and S were separated from SR and RS. 

Methyl palmitate, mono (2-ethylhexyl) phthalate, methyl myristate, palmitic acid, methyl laurate, eugenol and nicotine were rich in R root exudates (R/S > 1.6), while 4-hydroxybenzoic acid and sorbitol were abundant in S (S/R > 1.6). Dipropyl phthalate, dibutyl phthalate and octadecanoic acid were only detected in R and grafting varieties, while 4-tert-butylphenol and 2,6-di-tert-butylphenol were only found in S root exudates and grafting varieties. More compounds were detected in root exudates of the grafted cultivars than in the stock parent and scion parent, with 21 and 29 compounds in RS and SR, respectively. Most of them were hydrocarbons and benzene. Grafting also induced the change in the relative content (indicated as peak area percentage) of compounds. Most compounds with relatively high content in R were lower in grafted cultivars but higher than that in S. Moreover, most of these compounds in SR were higher than those in RS except for nicotine. Dipropyl phthalate, dibutyl phthalate, methyl laurate, and octadecanoic acid were only found in SR and its rootstock parents. The compounds with high relative content in S showed the same trend. These results suggested that rootstock had more effect on those differential compounds than scion. 

### 3.3. Allelopathy of Root Exudates on Phytophthora nicotianae

**Figure 4:**
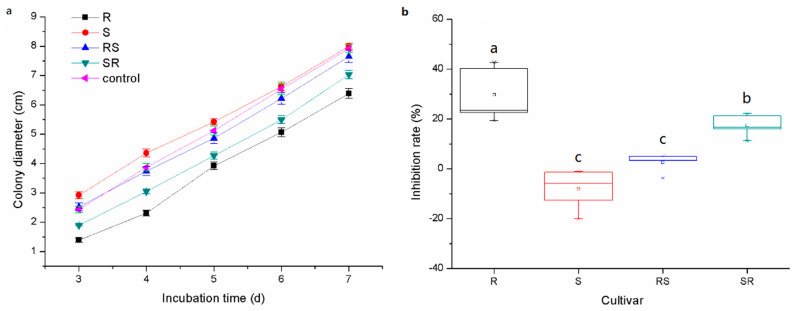
The effect of tobacco root exudates on mycelium growth of *Phytophthora nicotianae*. R and S represent resistant and susceptible cultivars; SR and RS represent different scion/stock combinations. The error bars in line chart (**a**) represent standard error of the mean. The border, horizontal line, upper and lower tentacles of box-plot (**b**) represent the interquartile range (IQR), median value, 1.5 times IQR range beyond the upper and lower quartiles, respectively; lower case letters indicate statistical differences (*p* < 0.05).

The effects of root exudates on *P. nicotianae* mycelium growth varied for the different cultivars tested. The mycelium growth treated with different root exudates began to show significant differences on the third day of treatment (Figure 4a, Figure S1). Compared with control, root exudates of S displayed a certain stimulation on *P. nicotianae* mycelium growth with a promotion rate of 1.01–20.16% (Figure 4). Root exudates of R and SR showed significant inhibitory effects (*p* < 0.05) with inhibition rates of 19.32–42.80% and 11.24–22.22%, respectively. Compared to the control, no obvious effect was observed in RS. It can be seen that the allelopathy of root exudates was associated with the disease resistance of the cultivars. Root exudates from the disease-resistant cultivar inhibited the mycelium growth of *P. nicotianae*, while root exudates from the susceptible cultivar stimulated mycelium growth. The allelopathy of root exudates was affected by grafting, and more greatly influenced by rootstocks. The results of correlation analysis showed that the mycelium inhibitory activity had a significant correlation with the resistance of the cultivars and grafting materials (R^2^ = 0.6673).

**Figure 5:**
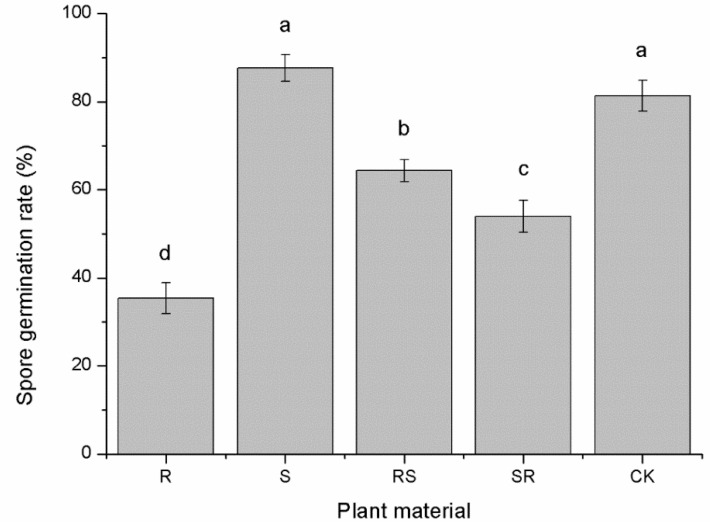
Influence of root exudates from different resistant tobaccos on zoospore germination of *P. nicotianae.* R and S represent resistant and susceptible cultivars; SR and RS represent different scion/stock combinations. Lower case letters indicate statistical differences (*p* < 0.05); the error bars represent standard error of the mean.

Similar trends were also noted for the effects of root exudates on *P. nicotianae* zoospore germination. As shown in Figure 5, there were obvious differences in the effect of root exudates on zoospore germination of *P. nicotianae* among cultivars (Figure 4). Germination of zoospores treated with root exudates of R and its grafting combinations (RS and SR) was significantly inhibited. R showed the strongest inhibitory effect with an inhibition rate of 56.56%, followed by SR with an inhibition rate of 33.61%. Zoospore germination was greatest for zoospores treated with root exudates of S, but there was no statistical difference between zoospores treated with S root exudates and the control. 

### 3.4. Allelopathy of Compounds on Phytophthora nicotianae Mycelium Growth

Esters, fatty acids, organic acids, phenols, alcohols, alkaloids and terpenes were known as possible allelochemicals. Therefore, 14 compounds belonging to the above types were selected to determine the allelopathy effect on *P. nicotianae*. They included six compounds only being detected in exudates of R (dipropyl phthalate, dibutyl phthalate, methyl laurate and octadecanoic acid) or S (4-tert-butylphenol and 2,6-di-tert-butylphenol), and eight compounds significantly differing between R and S (fold change of R/S > 1.5 or < 0.6) (Table S2). Most of the compounds tested showed a dose effect, i.e., as the concentration increased, the inhibition of the growth of *P. nicotianae* mycelia also increased (Table 1). At 5 μg/mL, only 4-tert-butylphenol and mono (2-ethylhexyl) phthalate exhibited a significant inhibitory effect compared to the DMSO control. Under treatment with high concentration (50 μg/mL and 500 μg/mL), nine compounds showed different inhibition ability. Eugenol, 4-tert-butylphenol and mono (2-ethylhexyl) phthalate displayed higher inhibitory effects, which reached more than 50% of inhibition rate by 50 μg/mL treatment, and completely suppressed the growth of *P. nicotianae* by 500 μg/mL treatment. A 500 μg/mL concentration of 4-hydroxybenzoic acid also completely inhibited the growth of *P. nicotianae*, but a 50 μg/mL concentration 4-hydroxybenzoic acid showed a weak inhibition rate (18.82%). Similar to 4-hydroxybenzoic acid, 2,6-di-tert-butylphenol, dipropyl phthalate and methyl myristate showed strong inhibitory effect (over 50% of inhibition rate) only at 500 μg/mL, while weak or no inhibitory ability were found for octadecanoic acid, hexadecanoic acid, nicotine, sorbitol, and methyl palmitate. A 50 μg/mL concentration of nicotine stimulated *P. nicotianae* mycelium growth significantly (*p* < 0.05) compared to DMSO. 

*P. nicotianae* mycelium grew better in sorbitol medium than in glucose medium (Table 2). Under relatively low osmotic potential (6.25 mM and 12.5 mM of solute), the effect of sorbitol on the growth of *P. nicotianae* was significantly higher than that of glucose (*p* < 0.05). High concentration of sorbitol (>50 mM) can be more effective than glucose to increase *P. nicotianae* mycelium growth.

## 4. Discussion

Grafting technology is widely used in the production of vegetables, fruits, and other important economic crops, and has been used to protect many important crops from soil-borne diseases [4,5,6,7]. The current study found that a resistant tobacco cultivar used as the rootstock or scion improved the disease resistance of the susceptible cultivar, and the difference in exudate composition might contribute to that. Our results are beneficial for filling the gap in understanding how grafting or using resistant cultivars can help reduce crop infection.

Accumulating evidence has demonstrated that root exudates are closely related to plant disease resistance. The root exudate composition can be species and genotype-specific, and these exudates can affect plant pathogenesis [24,25,26]. For example, root exudates secreted from tomato and pea, can promote mycelium growth and spore germination of *F. oxysporum* [27,28]. In contrast, root exudates from some disease-resistant varieties of lentils, capsicum, chickpea and cotton inhibited *F. oxysporum* [29,30]. These studies have aroused interest in selecting resistant plant varieties to study the effect of root exudates. Wu et al. [31] reported that root exudates of watermelon resistant varieties can inhibit the growth of *F. oxysporum* f. sp. *niveum*, while those of susceptible varieties promoted growth. Schalchli et al. [32] reported that the allelopathy of root exudates of different wheat varieties to *Gaeumannomyces grainis* var. *tritici* was significantly different. Similar results have been found in other crop-disease systems: egglant-*Verticillium dahliae* [18], faba bean-*F. oxysporum* f. sp. *fabae* [33], pepper-*Phytophthora capsici* [34], and cotton-*F. oxysporum* f.sp. *vasinfectum* [35]. Consistent with previous studies, the mycelium growth and spore germination of *P. nicotianae* were inhibited significantly by the root exudates of tobacco resistant cultivar Gexin 3 and its combination as a rootstock SR, while the root exudates of the susceptible cultivar Xiaohuangjin 1025 stimulated mycelium growth. The above results indicated that plant disease resistance can be partly manifested by the allelopathy of root exudates to pathogens. Generally, root exudates of resistant varieties inhibit the growth of pathogens, while those of susceptible varieties often show a stimulatory effect. However, it has also been reported that the root exudates of different plant resistant varieties had no significant effect in the allelopathy against pathogens [27]. Other studies have reported that although there are differences among varieties, root exudates can promote the growth of pathogens [36], suggesting the complex relationship between root exudates and plant resistance.

Interestingly, grafting not only changed the resistance of tobacco to black shank, but also the allelopathy of root exudates to pathogens, which showed that allelopathy was positively correlated with resistance (R^2^ = 0.6673). Irrespective of whether the resistant varieties were used as rootstock or scion, root exudates of the grafting cultivars showed greater inhibitory effects on *P. nicotianae* than the susceptible parent, but lower inhibitory effects than that of the resistant parent. Similarly, Ling et al. [19] reported that the root exudates of watermelon significantly inhibited *F. oxysporum* f.sp. *niveum* after rootstock grafting. Duan et al. [13] found that root exudates of resistant rootstocks and grafted pepper could inhibit the growth of *Fusarium solani* and *Ralstonia solanacearum*, compared with root exudates of self-rooted capsicum. Zhao et al. [37] reported that root exudates from bacterial wilt resistant rootstocks and grafted tomatoes could significantly inhibit the growth of *R. solanacearum*. These results indicated that allelopathy of root exudates was one of the important mechanisms for grafting disease-resistant rootstock to control soil-borne diseases.

The chemical components of root exudates are complex and diverse, including primary metabolites such as sugars, amino acids, and organic acids, as well as secondary metabolites such as flavonoids, glucosides, and auxin. So far, more than 10,000 plant root exudates have been identified [38], mainly including hydrocarbons, esters, phenols, phenolic acids, aldehydes, etc. [39]. Yu et al. [40] and Deng et al. [41] identified root exudates of Burley, K326, NC89 and other tobacco varieties by GC-MS, and found that root exudates mainly include amino acids, organic acids, phenolic acids, fatty acids, sterols, and other organic compounds. Different varieties, growth periods, and collection conditions also have a major impact on the concentration and composition of root exudates. We also found that grafting increased the species of root exudates and changed the relative content of chemical components, and root exudates from grafted cultivar showed more similarity with its rootstock parent. These results indicated that the abundance or composition of the exudates may have a unique effect on the pathogen. 

The different allelopathy to pathogens is caused by particular compounds in root exudates, some of which can obviously inhibit the growth or spore germination of soil-borne pathogens [26], while others stimulated growth or spore germination [42]. Hao et al. [26] found that ferulic acid from watermelon root exudates had a stimulatory effect on *F. oxysporum* growth, and identified it as the key component in the occurrence of continuous cropping fusarium wilt. Tian et al. [43] reported that ferulic acid and coumaric acid from strawberry root exudates significantly promoted the occurrence of *Anthracnose* crown rot. Our results showed that root exudates of a resistant cultivar contained more compounds with inhibitory effects on *P. nicotianae* growth. Among the four compounds with the strongest inhibitory activity, 4-tert-butylphenol and p-hydroxybenzoic acid were rich in the root exudates of susceptible varieties. In general, the species and relative content of inhibitory compounds were more abundant in disease-resistant cultivars. Most of the above compounds showed convergence between grafted cultivar and its rootstock parent, indicating that these compounds were closely related to plant disease resistance.

The relative content of sorbitol in root exudates of susceptible varieties is high, which may be closely related to tobacco susceptibility. Sorbitol is a polyol, which is believed to have multiple biological activities and play an important role in the germination and appressorium formation of fungal spores [44,45]. However, unlike fungi, the conidia of oomycetes contain no polyols [46], but it has been reported that exogenous sorbitol can promote the germination of zoospores and formation of germ tubes [47]. Our results show that sorbitol is more beneficial to the growth of *P. nicotianae* flagella under the condition of iso-osmosis. It is speculated that sorbitol may play a role in the germination of zoospores and the elongation of germ tubes of *P. nicotianae*, but it needs further experimental confirmation. In addition, when the zoospores of *Phytophthora* are released, chemotaxis is needed to find the host and initiate the infection process [48]. Therefore, chemotaxis is very important for *Phytophthora* infection and is considered to be related to plant disease resistance [49]. Sorbitol is also an important chemotactic factor in root exudates. The chemical gradient produced by sorbitol can promote the movement of many microorganisms towards plants [50,51]. Whether sorbitol also has chemotaxis to *P. nicotianane* zoospores remains to be tested.

It should be noted that the discovery of the activity of these compounds can only partially explain their relationship with plant disease resistance. Root exudates or their active components can also affect plant growth, soil physical and chemical properties and microbial community structure, indirectly affecting the occurrence and development of diseases [52,53,54]. Among 14 different compounds in root exudates of susceptible and resistant varieties, 4-tert-butylphenol, p-hydroxybenzoic acid, 2,6-di-tert-butylphenol and eugenol are phenolic acids. The accumulation of these compounds in the soil can produce plant autotoxicity, promote the propagation of pathogenic bacteria, lead to an imbalance of soil microflora and reduce soil enzyme activity and nutrient content, etc. These factors are considered to be important factors causing continuous cropping obstacles [52]. P-hydroxybenzoic acid, lauric acid, myristic acid and palmitic acid have been proved to be related to continuous cropping disorders of tobacco [55,56]. 2,4-di-tert-butylphenol was reported to promote the occurrence of Lily wilt [57], while 2,6-di-tert-butylphenol and eugenol were reported to have autotoxic effects on wheat [58], peanut [59], and pepper [60]. The content of phenolic acids in root exudates of susceptible cultivars was higher than that of resistant cultivars, suggesting that these substances might be related to the susceptibility of cultivars.

## 5. Conclusions

Grafting on disease-resistant rootstock can effectively improve the resistance of tobacco to black shank, which is closely related to the allelopathy of root exudates. The root exudates from resistant cultivar significantly inhibited the growth of *P. nicotianae*, while root exudates from susceptible cultivars promoted growth of *P. nicotianae*, and the effect of root exudates from the reciprocal grafts were intermediate between their graft parents. The relative content of esters was rich in resistant varieties, while hydrocarbons and phenolic acids were high in susceptible varieties. The species and content of inhibitory components in root exudates of the resistant cultivar were more than that of the susceptible cultivar. Eugenol, p-hydroxybenzoic acid, 4-tert-butylphenol, p-hydroxybenzoic acid, 2-ethylhexyl-phthalate, and sorbitol were the potential allelochemicals on *P. nicotianae*. It is speculated that there is a relation between sorbitol and susceptibility of tobacco to *P. nicotianae*. 

It should be noted that the mechanism of root exudates affecting plant disease resistance is very complex. This study only preliminarily revealed that the allelopathy of root exudates is closely related to tobacco disease resistance. Further analysis of the complex networks of root exudates and plant disease control techniques is expected to provide new strategies, techniques, and approaches for disease control.

## Figures and Tables

**Table 1:** Effect of tested compounds on mycelium growth of *P. nicothianae*.

Compound	Diameter (cm)			Inhibition Rate (%)		
	5 μg/mL	50 μg/mL	500 μg/mL	5 μg/mL	50 μg/mL	500 μg/mL
p-tert-butylphenol	3.68 ± 0.20 e	0.22 ± 0.03 g	0 i	13.00 a	94.82 a	100.00 a
4-hydroxybenzoic acid	4.25 ± 0.09 b	3.45 ± 0.05 d	0 i	−0.47 d	18.82 e	100.00 a
mono (2-ethylhexyl) phthalate	3.65 ± 0.05 e	1.78 ± 0.13 f	0 i	13.71a	58.12 b	100.00 a
lauric acid	4.28 ± 0.23 b	2.53 ± 0.08 e	0.67 ± 0.06 h	−1.18 d	40.47 d	83.78 b
2,6-bis (1,1-dimethylethyl) phenol	3.92 ± 0.19 d	3.92 ± 0.20 c	1.15 ± 0.05 g	7.33 b	7.76 h	72.15 c
2-benzenedicarboxylic acid dipropyl ester	4.32 ± 0.10 ab	3.75 ± 0.17 cd	1.42 ± 0.08 f	−2.13 d	11.76 f	65.62 d
tetradecanoic acid	4.38 ± 0.03 a	4.05 ± 0.05 bc	1.88 ± 0.13 e	−3.55 d	4.71 i	54.48 e
dibutyl phthalate	4.32 ± 0.24 a	4.15 ± 0.25 b	3.88 ± 0.03 c	−2.13 d	2.35 j	6.05 h
methyl palmitate	4.45 ± 0.13 a	4.42 ± 0.20 a	4.3 ± 0.40 ab	−5.20 e	−4.00 k	−4.12 j
nicotine	4.18 ± 0.12 bc	4.98 ± 0.03 a	4.42 ± 0.10 a	1.18 d	−17.18 l	−7.02 k
eugenol	4.05 ± 0.05 c	2.07 ± 0.03 f	0 i	4.26 c	51.29 c	100.00 a
sorbitol	4.26 ± 0.11 b	4.37 ± 0.10 a	4.05 ± 0.08 b	−0.71 d	−2.82 k	1.94 i
octadecanoic acid	4.31 ± 0.0.06 a	3.96 ± 0.07 c	3.34 ± 0.05 d	−1.89	6.82 h	19.13 g
n- hexadecanoic acid	4.28 ± 0.10 b	3.85 ± 0.09 c	2.92 ± 0.10 d	−1.18	9.41 g	29.30 f
DMSO (control)	4.23 ± 0.06 bc	4.25 ± 0.09 b	4.13 ± 0.15 b	-	-	-

Values are the means of three replicates ± SD; different letters indicate statistically significant differences between the treatments (*p* < 0.05).

**Table 2:** The effects of glucose and sorbitol on *P. nicotianae* mycelium growth.

Compound	Mycelium Diameter (cm)				
	6.25 mM	12.5 mM	25 mM	50 mM	100 mM
glucose	5.28 ± 0.16 b	5.20 ± 0.20 b	5.68 ± 0.16 a	5.52 ± 0.23 b	5.37 ± 0.23 a
sorbitol	5.90 ± 0.23 a	5.80 ± 0.18 a	5.62 ± 0.08 a	5.90 ± 0.05 a	5.63 ± 0.21 a

Values are the mean ± SD of three replicates; values within a column followed by different letters are significantly different (*p* < 0.05).

## Supplementary Materials

The following are available online at https://www.mdpi.com/2223-7747/9/12/1652/s1, Figure S1: The effect of tobacco root exudates on mycelium growth of *Phytophthora nicotiana* (the 3rd day after culture), Table S1: Root exudates in different tobacco variety and grafting treatment, Table S2: Discriminant metabolites in tobacco root exudates based on GC-MS analysis.

## References

[1] Panabieres F., Ali G.S., Allagui M.B., Dalio R.J.D., Gudmestad N.C., Kuhn M., Roy S.G., Schena L., Zampounis A. (2016). *Phytophthora nicotianae* diseases worldwide: New knowledge of a long-recognised pathogen. Phytopatho. Mediterr..

[2] Wang Y., Wei K., Han X., Zhao D., Zheng Y., Chao J., Gou J., Kong F., Zhang C.S. (2019). The Antifungal Effect of Garlic Essential Oil on *Phytophthora nicotianae* and the Inhibitory Component Involved. Biomolecules.

[3] Wang H.C., Chen X.J., Cai L.T., Cao Y., Lu N., Xia H.Q., Wang M.S., Shang S.H. (2013). Race distribution and distribution of sensitivities to mefenoxam among isolates of *Phytophthora parasitica* var. *nicotianae* in Guizhou province of China. Crop Prot..

[4] Gilardi G., Gullino M.L., Garibaldi A. (2013). Critical aspects of grafting as a possible strategy to manage soil-borne pathogens. Sci. Hortic..

[5] Abebe A.M., Wai K.P.P., Siddique M.I., Mo H., Yoo J.Y., Jegal Y., Byeon S., Jang K., Jeon S., Hwang J. (2016). Evaluation of *Phytophthora* root rot- and bacterial wilt-resistant inbred lines and their crosses for use as rootstocks in pepper (*Capsicum annuum* L.). Hortic. Environ. Biotechnol..

[6] Haegi A., De Felice S., Scotton M., Luongo L., Belisario A. (2017). *Fusarium oxysporum* f.sp. *melonis*-melon interaction: Effect of grafting combination on pathogen gene expression. Eur. J. Plant. Pathol..

[7] Mackey M., Kurosky A., Nazar R.N. (2018). A Graft Mimic Strategy for Verticillium Resistance in Tomato. Mol. Biotechnol..

[8] Schwarz D., Rouphael Y., Colla G., Venema J.H. (2010). Grafting as a tool to improve tolerance of vegetables to abiotic stresses: Thermal stress, water stress and organic pollutants. Sci. Hortic..

[9] Wang H., Shi J., Luo Z., Chen Y., Zhang Y., Li Z. (2018). Resistant identification of tobacco grafted seedlings to black shank. Plant Prot..

[10] Liu J., Zhu B., Hu S., Wang C., Yang T. (2018). Effects of grafting on growth, development and black shank resistance of flue-cured tobacco. Tob. Sci. Technol..

[11] Wu F., An M. (2011). Effects of Watermelon Cultivars with Different Resistances to Fusarium oxysporum f.sp. niveum and Grafting on Rhizosphere Soil Microorganism Population and Community Structure. Chin. Agric. Sci..

[12] Guan W., Zhao X., Hassell R., Thies J.A. (2012). Defense mechanisms involved in disease resistance of grafted vegetables. HortScience.

[13] Duan X., Bi H.G.T., Li G.X., Wu Q., Li M., Ai X.Z. (2017). Root characteristics of grafted peppers and their resistance to Fusarium solani. Biol. Plant..

[14] Philippot L., Raaijmakers J.M., Lemanceau P., Van Der Putten W.H. (2013). Going back to the roots: The microbial ecology of the rhizosphere. Nat. Rev. Microbiol..

[15] Baetz U., Martinoia E. (2014). Root exudates: The hidden part of plant defense. Trends Plant Sci..

[16] Huang X., Chaparro J.M., Reardon K.F., Zhang R., Shen Q., Vivanco J.M. (2014). Rhizosphere interactions: Root exudates, microbes, and microbial communities. Botany.

[17] De-la-Pena C., Badri D.V., Lei Z., Watson B.S., Brandao M.M., Silva-Filho M.C., Sumner L.W., Vivanco J.M. (2010). Root secretion of defense-related proteins is development-dependent and correlated with flowering time. J. Biol. Chem..

[18] Zhou B., Chen Z., Du L., Xie Y., Ye X. (2011). Allelopathy of the root exudates from different resistant eggplants to verticillium wilt (Verticillium dahliae Kleb). Acta Ecol. Sin..

[19] Ling N., Zhang W., Wang D., Mao J., Huang Q., Guo S., Shen D. (2013). Root Exudates from Grafted-Root Watermelon Showed a Certain Contribution in Inhibiting *Fusarium oxysporum* f. sp. *niveum*. PLoS ONE.

[20] Zhang C., Gao J., Han T., Tian X., Wang F. (2017). Integrated control of tobacco black shank by combined use of riboflavin and Bacillus subtilis strain Tpb55. BioControl.

[21] Lee J.M., Kubota C., Tsao S.J., Bie Z., Echevarria P.H., Morra L., Oda M. (2010). Current status of vegetable grafting: Diffusion, grafting techniques, automation. Sci. Hortic..

[22] Zhang C., Feng C., Zheng Y., Wang J., Wang F. (2020). Root Exudates Metabolic Profiling Suggests Distinct Defense Mechanisms Between Resistant and Susceptible Tobacco Cultivars Against Black Shank Disease. Front. Plant Sci..

[23] Badri D.V., De-La-Peña C., Lei Z., Manter D.K., Chaparro J.M., Guimarães R.L., Sumner L.W., Vivanco J.M. (2012). Root Secreted Metabolites and Proteins Are Involved in the Early Events of Plant-Plant Recognition Prior to Competition. PLoS ONE.

[24] Dixon R.A. (2001). Natural products and plant disease resistance. Nature.

[25] Bertin C., Yang X., Weston L. (2003). The role of root exudates and allelochemicals in the rhizosphere. Plant Soil.

[26] Hao W.Y., Ren L.X., Ran W., Shen Q.R. (2010). Allelopathic effects of root exudates from watermelon and rice plants on *Fusarium oxysporum* f.sp. *niveum*. Plant Soil.

[27] Whalley W.M., Taylor G.S. (1973). Influence of pea-root exudates on germination of conidia and chlamydospores of physiologic races of Fusarium oxysporum f. pisi. Ann. Appl. Biol..

[28] Steinkellner S., Mammerler R., Vierheilig H. (2008). Germination of *Fusarium oxysporum* in root exudates from tomato plants challenged with different *Fusarium oxysporum* strains. Eur. J. Plant Pathol..

[29] Naqvi S.M.A., Chauhan S.K. (1980). Effect of root exudates on the spore germiantion of rhizosphere and rhizoplane mycoflora of chilli (*Capsicum annuum* L.) cultivars. Plant Soil.

[30] Stevenson P.C., Padgham D.E., Haware M.P. (1995). Root exudates associated with the resistance of four chickpea cultivars (*Cicer arietinum*) to two races of *Fusarium oxysporum* f.sp. *cicero*. Plant Pathol..

[31] Wu H., Liu D., Ling N., Bao W., Ying R., Shen Q. (2008). Influence of Root Exudates of Watermelon on *Fusarium oxysporum* f. sp. *niveum*. Soil Sci. Soc. Am. J..

[32] Schalchli H., Pardo F., Hormazabal E., Palma R., Guerrero J., Bensch E. (2012). Antifungal activity of wheat root exudate extracts on *Gaeumannomyces graminis* var. *tritici* growth. J. Soil Sci. Plant Nutr..

[33] Dong Y., Dong K., Zheng Y., Yang Z., Tang L., Xiao J. (2014). Allelopathic effects and components analysis of root exudates of faba bean cultivars with different degrees of resistance to *Fusarium oxysporum*. Chin. J. Eco-Agric..

[34] Wang J., Lyu Y., Yu D., Zhang W., Piao F., Shen S. (2014). Effects of Root Exudates from Different Resistant Pepper Varieties on *Phytophthora capsici*. China Veget..

[35] Ren Z., Gai Q. (2016). Root Exudates of Resistant and Susceptible Cotton Cultivars Sand lts Effects on *Fusarium oxysporum* f. sp. *vasinfectum*. Acta Agric. Boreali-Occident. Sin..

[36] Li X., Liu B., Heia S., Liu D., Han Z., Zhou K., Cui J., Luo J., Zheng Y. (2009). The effect of root exudates from two transgenic insect-resistant cotton lines on the growth of *Fusarium oxysporum*. Transgenic Res..

[37] Zhao W., Zheng X., Zhang Y., Zhong C., Yang Y., Yu W. (2019). Effects of Root Exudates from Tomato Grafted with Different Rootstocks on *Ralstonia solanacearum* and Seedling Growth. China Veget..

[38] Weston L.A., Ryan P.R., Watt M. (2012). Mechanisms for cellular transport and release of allelochemicals from plant roots into the rhizosphere. J. Exp. Bot..

[39] Scavo A., Abbate C., Mauromicale G. (2019). Plant allelochemicals: Agronomic, nutritional and ecological relevance in the soil system. Plant Soil.

[40] Yu H., Shen G., Gao X. (2013). Determination of tobacco root exudates by GC-MS. Acta Tab. Sin..

[41] Deng J., Zhang S., Zhang F., Zhang Y., Hu F., Li H. (2017). Autotoxins exuded from roots and the effects of PAEs on antioxidant capacity in roots of tobacco seedlings. Acta Ecol. Sin..

[42] Yang R.X., Gao Z.G., Liu X., Yao Y., Cheng Y. (2014). Root exudates from muskmelon (*Cucumis melon.* L) induce autotoxicity and promote growth of *Fusarium oxysporum* f. sp. *Melonis*. Allelopath. J..

[43] Tian G., Bi Y., Sun Z., Zhang L. (2015). Phenolic acids in the plow layer soil of strawberry fields and their effects on the occurrence of strawberry anthracnose. Eur. J. Plant Pathol..

[44] Tereshina V.M., Memorskaya A.S., Morozova E.V., Feofilova E.P. (2000). Alterations in the carbohydrate composition of the cytosol of fungal spores caused by temperature variations and the storage process. Microbiology.

[45] Feofilova E.P., Ivashechkin A.A., Alekhin A.I., Sergeeva Y.E. (2012). Fungal spores: Dormancy, germination, chemical composition, and role in biotechnology (review). Appl. Biochem. Microbiol..

[46] Pfyffer G.E., Boraschi Gaia C., Weber B., Hoesch L., Orpin C.G., Rast D.M. (1990). A further report on the occurrence of acyclic sugar alcohols in fungi. Mycol. Res..

[47] Campbell A.M., Moon R.P., Duncan J.M., Gurr S.J., Kinghorn J.R. (1989). Protoplast formation and regeneration from sporangia and encysted zoospores of *Phytophthora infestans*. Physiol. Mol. Plant Pathol..

[48] Morris E.P.F., Ward W.B. (1992). Chemoattraction of zoospores of the soybean pathogen, *Phytophthora sojae*, by isoflavones. Physiol. Mol. Plant Pathol..

[49] Zhang Z., Xu Y., Song G., Gao X., Zhao Y., Jia M., Chen Y., Suo B., Chen Q., Wu D. (2019). *Phytophthora sojae* zoospores differ in chemotaxis to the root and root exudates of host soybean and nonhost common bean. J. Gen. Plant Pathol..

[50] Hsu S.T., Huang H.C. (1987). Chemotaxes of *Erwinia carotovora* Subsp. *carotovora* and *Erwinia chrysanthemi*. Curr. Plant Sci. Biotechnol. Agric..

[51] Hu X., Xie L., Yu C., Li Y., Liu S., Zhang C., Liao X. (2011). Chemotaxis of *Bacillus megaterium* strain A6 towards organic acid and saccharide from roots exudates of rapeseed. Chin. J. Oil Crop Sci..

[52] Richardson A.E., Lynch J.P., Ryan P.R., Delhaize E., Smith F.A., Smith S.E., Harvey P.R., Ryan M.H., Veneklaas J.E., Lambers H. (2011). Plant and microbial strategies to improve the phosphorus efficiency of agriculture. Plant Soil.

[53] Yuan J., Wu Y., Zhao M., Wen T., Huang Q., Shen Q. (2018). Effect of phenolic acids from banana root exudates on root colonization and pathogen suppressive properties of *Bacillus amyloliquefaciens* NJN-6. Biol. Control.

[54] Li X., Ding C., Hua K., Zhang T., Zhang Y., Zhao L., Yang Y., Liu J., Wang X. (2014). Soil sickness of peanuts is attributable to modifications in soil microbes induced by peanut root exudates rather than to direct allelopathy. Soil Biol. Biochem..

[55] Zhang K., Xu T., Shen F., Shi B., Gu M., Shou A., Li X. (2013). Phenolic Acids in *Nicotiana tobacco* L. Root Exudate and Their Autotoxicity Effects. Southwest China J. Agric. Sci..

[56] Yu H., Song X., Wang S., Cao L., Guo L., Wang X., Peng G. (2015). Effects of Low Molecular Weight Organic Acids on Soil Enzymes Activities and Bacterial Community Structure. Chin. Agric. Sci..

[57] Huang W. (2018). Study on Synergetic Effect of 2,4-DTBP and Fusarium on the Occurrence of Fusarium Wilt in Lanzhou Lily. J. Gansu Agric. Univ..

[58] Zhao C., Zhou H., Chai Q., Huang G., Liu H., Zhu J. (2014). Effects of eugenol and intercropped faba-bean on wheat root growth under different water supply conditions. Acta Pratacult. Sin..

[59] Liu P., Zhao H., Tang Z., Zhang Y., Lin H., Shen Y., Wsng J., Wan S. (2015). Effects of continuous cropping on root exudates of different resistance peanut (*Arachis hypogaea* L.) varieties and allelochemicals content in soil. Chin. J. Oil Crop Sci..

[60] Sun M., Ren X., Yao H., Cai W., Li M. (2017). Allelopathic effect of 2, 6-Di-tert-butylphenol on pepper and its alleviating substances. Jiangsu Agric. Sci..

