# Supplementary material for: Rootstock-Scion Interaction Affects the Composition and Pathogen Inhibitory Activity of Tobacco (Nicotiana tabacum L.) Root Exudates
Source: Plants (Basel). 2020 Nov 26;9(12):1652. doi: 10.3390/plants9121652 (PMC7760518; doi:10.3390/plants9121652)
Supplement: Supplementary file 1 [file plants-09-01652-s001.pdf]

plants

MDPI

Supplementary Materials

Rootstock-Scion Interaction Affects the Composition and Pathogen Inhibitory Activity of Tobacco (*Nicotiana tabacum* L.) Root Exudates

Cheng-Sheng Zhang [1,2,*,†], Yanfen Zheng [1,2,†], Lijuan Peng [3] and Jianmin Cao [1,*]

[1] Tobacco Research Institute of Chinese Academy of Agricultural Sciences, Qingdao 266101, China; zhengyanfen@caas.cn
[2] Special Crops Research Center of Chinese Academy of Agricultural Sciences, Qingdao 266101, China
[3] Yunnan Tobacco Quality Supervision and Test Station, Kunming 650106, China; pengljkm2003@hotmail.com
* Correspondence: zhchengsheng@126.com (C.-S.Z.); caojianmin@caas.cn (J.C.); Tel.: +86-0532-8870-2115 (C.-S.Z.)
† These authors contributed equally to this work.

Table S1. Root exudates in different tobacco variety and grafting treatment.

Type	Compound name	R	S	RS	SR
hydrocarbons	n-dodecane	-	-	5.78±0.84	-
	tridecane	6.81±0.05	8.46±0.05	9.55±0.12	-
	tetradecane	3.75±0.03	9.27±0.11	3.69±0.06	-
	n-pentadecane	-	-	-	1.12±0.02
	n-heptadecane	-	-	-	1.76±0.04
	octadecane	-	-	8.43±0.11	3.18±0.05
	n-nonadecane	-	-	-	0.91±0.04
	n-eicosane	-	6.31±0.10	-	1.55±0.06
	n-henicosane	-	-	-	1.23±0.02
	9-octyl-heptadecane	-	-	-	4.04±0.03
	n-hexacosane	-	-	-	4.16±0.07
	heptacosane	-	-	-	5±0.13
	1-iodohexadecane	-	-	-	3.96±0.17
benzene	1,3-diphenyl-1-butene	8.96±0.22	8.18±0.35	11.96±0.47	-
	1,2,4,5-tetramethylbenzene	14.79±0.62	19.9±0.85	6.74±0.23	-
	1,3-dimethyl-benzene	10.26±0.56	13.32±0.93	6.74±0.42	15.01±1.01
	1-ethyl-3-methyl-benzene	12.43±0.74	-	-	11.76±0.61
	1,2,3-trimethylbenzene	-	-	12.78±1.05	13.57±0.82
ester	methyl palmitate	5.05±0.33	2.35±0.14	3.24±0.26	2.63±0.18
	methyl oleate	2.75±0.12	3.26±0.09	2.98±0.31	3.31±0.13
	dipropyl phthalate	1.12±0.07	-	-	0.91±0.06
	mono(2-ethylhexyl) phthalate	3.32±0.04	1.14±0.05	1.88±0.02	2.68±0.05
	dibutyl phthalate	2.29±0.10	-	-	1.14±0.09
	methyl myristate	4.55±0.35	1.46±0.06	1.99±0.07	3.12±0.20
	methyl Laurate	4.21±0.13	-	1.88±0.25	1.14±0.06
phenol	eugenol	3.45±0.05	2.21±0.03	2.78±0.11	3.1±0.16
	4-tert-butylphenol	-	3.78±0.05	2.1±0.04	1.08±0.07
	2,4-di-tert-butylphenol	-	5.93±0.12	2.09±0.06	0.99±0.07
fatty acid	n-hexadecanoic acid	4.66±0.18	2.43±0.14	3.39±0.22	3.87±0.33
	octadecanoic acid	1.92±0.07	-	1.25±0.05	1.05±0.03
organic acid	4-hydroxybenzoic acid	0.84±0.06	2.23±0.11	0.58±0.05	1.98±0.09
terpene	camphor	1.13±0.06	1.03±0.05	-	1.78±0.11
alcohol	sorbitol	2.66±0.17	6.43±0.36	3.39±0.19	0.87±0.23
alkaloid	nicotine	5.05±0.26	2.31±0.15	6.78±0.46	3.10±0.37

R, resistant cultivar; S, susceptible cultivar; SR, resistant cultivar as rootstock; RS, susceptible cultivar as rootstock; Value, peak area percentage. “-“, not detected.

Table S2. Discriminant metabolites in tobacco root exudates based on GC-MS analysis.

Compounds	R/S	S/RS	R/SR
methyl palmitate	2.15	0.73	1.92
mono(2-ethylhexyl) phthalate	2.91	0.61	1.24
methyl myristate	3.12	0.73	1.46
eugenol	1.56	0.79	1.11
n-hexadecanoic acid	1.92	0.72	1.20
4-hydroxybenzoic acid	0.38	3.84	0.42
sorbitol	0.41	1.90	3.06
nicotine	2.19	0.34	1.63

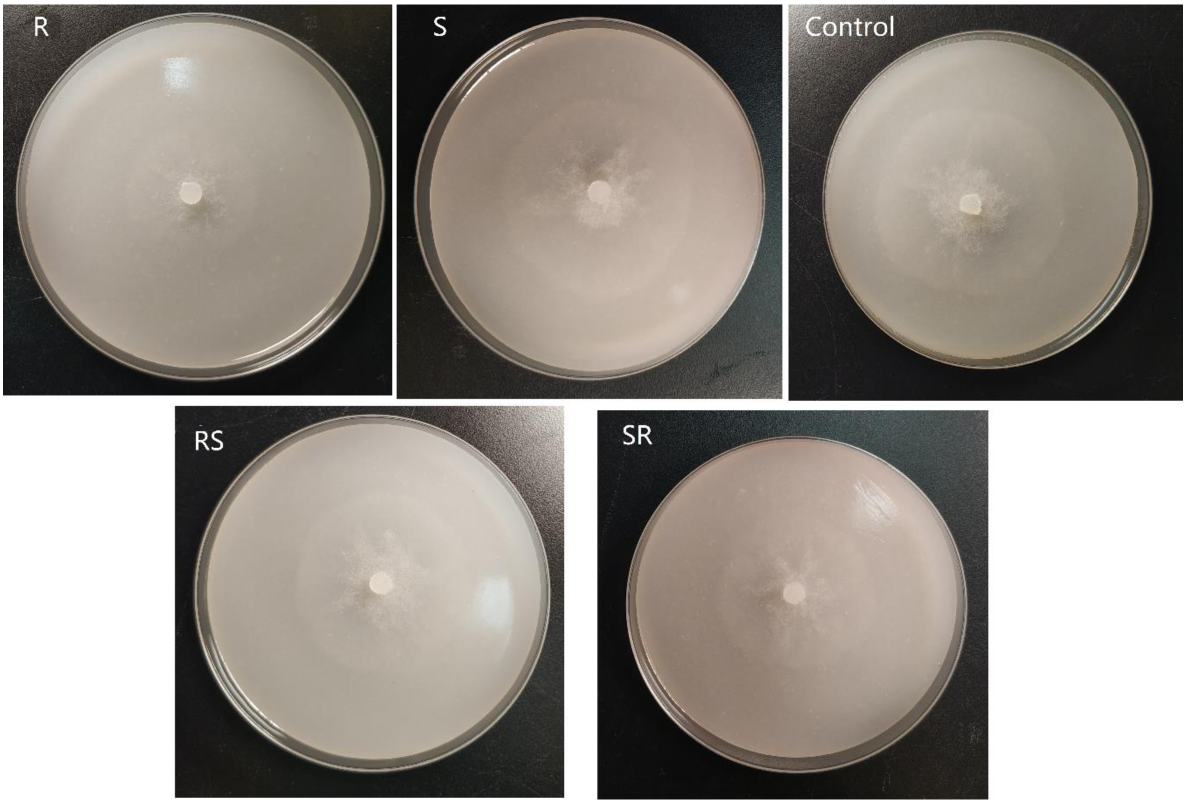

Figure S1. The effect of tobacco root exudates on mycelium growth of *Phytophthora nicotiana* (the 3rd day after culture). R and S represent resistant and susceptible cultivar; SR and RS represent different scion/stock combination.
